# Quantitative secondary electron imaging for work function extraction at atomic level and layer identification of graphene

**DOI:** 10.1038/srep21045

**Published:** 2016-02-16

**Authors:** Yangbo Zhou, Daniel S Fox, Pierce Maguire, Robert O’Connell, Robert Masters, Cornelia Rodenburg, Hanchun Wu, Maurizio Dapor, Ying Chen, Hongzhou Zhang

**Affiliations:** 1 School of Physics and CRANN, Trinity College Dublin, Dublin 2, Ireland; 2 Department of Materials Science and Engineering, the University of Sheffield, Sir Robert Hadfield Building, Mappin Street, Sheffield S1 3JD,United Kingdom; 3 Key Laboratory of Cluster Science of Ministry of Education, School of Physics, Beijing Institute of Technology, Beijing, 100081, People’s Republic of China; 4 European Centre for Theoretical Studies in Nuclear Physics and Related Areas (ECT*-FBK) and Trento Institute for Fundamental Physics and Applications (TIFPA-INFN), via Sommarive 18, Trento I-38123, Italy; 5 Institute for Frontier Materials, Deakin University, Waurn Ponds, Victoria 3216, Australia

## Abstract

Two-dimensional (2D) materials usually have a layer-dependent work function, which require fast and accurate detection for the evaluation of their device performance. A detection technique with high throughput and high spatial resolution has not yet been explored. Using a scanning electron microscope, we have developed and implemented a quantitative analytical technique which allows effective extraction of the work function of graphene. This technique uses the secondary electron contrast and has nanometre-resolved layer information. The measurement of few-layer graphene flakes shows the variation of work function between graphene layers with a precision of less than 10 meV. It is expected that this technique will prove extremely useful for researchers in a broad range of fields due to its revolutionary throughput and accuracy.

Work function plays an important role in high-performance electronic devices such as transistors^1^, gas sensors^2^, solar cells^3^ and field emitters^4^. It needs to be tailored to particular device applications for optimised performance (e.g. efficiency in solar cells), therefore work function engineering is an important step in the transition from lab-scale devices to industrial applications^5^. In two-dimensional (2D) few-layer materials (e.g. graphene) the work function is tuneable by varying the number of layers (thickness). A change in thickness of just one atomic layer can result in variations of several tens of meV^6^,^7^,^8^,^9^. Other important parameters in work function engineering in such materials are chemical doping, self-assembled monolayer (SAM) treatment and strain^5^,^10^,^11^,^12^ and the substrate material, including it’s local crystalline orientation or local oxidation and contaminants^13^. Therefore a simple method to detect the work function of graphene with high speed and atomic level accuracy is important. Several techniques have already been demonstrated for the characterisation of the work function of graphene^6^,^7^. However, the application of these techniques appears to be limited by various environmental factors and specimen interactions in scanning probe techniques^9^, such as low throughput/low spatial resolution for photoelectron emission spectroscopy (PEEM)^14^. Thus their applications are heavily restricted.

Secondary electron (SE) contrast is promising in surmounting these barriers to work function characterisation. SEs are excited by a charged beam of energetic particles, e.g. electrons or ions at the position where the beam enters the specimen, which might be used for structure modification^15^,^16^. They are exploited for surface imaging in charged particle beam scanning microscopes, such as the scanning electron microscope (SEM) and helium ion microscope (HIM)^17^,^18^,^19^,^20^. These microscopes provide quantitative measurement of surface dimensions with sub-nanometer resolution and intuitive image interpretation. The generation, emission, and detection of SEs varies with sample properties and the electromagnetic environment around the sample. These dependencies may offer mechanisms to evaluate sample properties in addition to lateral dimensions. For example, SEs need to overcome the surface potential in order to escape into vacuum, i.e., the work function. Since the work function limits the SE escaping from the sample, it is intuitive that SE imaging could be used to extract the work function. However, this is an extremely challenging task, since the image contrast may also be affected by other factors, such as beam parameters, beam-induced contamination, specimen electric potential, SE collection efficiency, etc. Quantitative measurements of work function based on SE imaging have been scarcely reported^21^,^22^, especially for graphene and other 2D materials. The attempt to extract their properties (e.g. thickness) from SE imaging has been reported and a thickness-dependent SE contrast has been confirmed^23^,^24^,^25^. However, inconsistency exists in these works (e.g. the layer-dependence for the SE contrast) and a quantitative approach to extract work function is still absent.

Here, we present a method to extract quantitative information from the SE contrast of graphene. We have established imaging parameters to acquire reproducible SE contrast from graphene samples. Atomic level identification of graphene thickness up to 10 layers is demonstrated. A model based on illumination by both the primary beam and backscattered particles was established to explain the observed layer-dependent SE contrast. Furthermore, by subtracting the contribution from SE attenuation, this model allows the quantitative determination of work function variations. The validity of the model is further supported by the obtained graphene SE energy spectra. Our work shows a high throughput, high resolution way to effectively determine the work function of graphene, and extends the application fields of SE contrast to quantitative analysis.

We first evaluate the visibility of graphene in the SE imaging and compare it with optical imaging. The images shown in Fig. 1a were taken from a few-layer graphene flake (on a Au substrate) using an optical microscope (OM), a SEM and a HIM, respectively. The flake is almost invisible in the optical image (Fig. 1a), while it is evident in the SE images captured by both SEM and HIM (Fig. 1b,c). We have found that few-layer graphene on other metal substrates (e.g., Cu and Ni) shows similar optical and SE visibilities (see Figure S1.1 in the supplementary information). To be able to see few-layer graphene through an OM, the reflective amplitude of the illumination light at the air/graphene/substrate interface must be significantly different from that at the air/substrate interface, which depends on both the wavelength of the light and the optical behaviour (i.e. the reflective coefficient) of the substrates^26^,^27^. This means the graphene is visible for a restricted combination of substrates and light. Monoatomic metallic substrates cannot produce such differences in the amplitude of the reflected light, which results in the low optical contrast for the whole visible light range (see detailed discussions in section 2 of the supplementary information). However, graphene flakes are discernible in SE images regardless of the type of primary beam (electrons or He^+^ ions) or the substrates.

The contrast of a graphene flake in a SE image must be larger than c.a. 2% to be visible. The contrast (C_g_) is defined as the intensity difference between graphene (I_g_) and its substrate (I_s_): *C*_*g*_ = (*I*_*g*_/*I*_*g*_−1)×100%. The contrast varies with imaging conditions. For example, Fig. 1b shows SEM images of few-layer graphene on a highly p-doped Si substrate covered with 285 nm SiO_2_ (SiO_2_/Si). They were acquired with electron beam energies ranging from 0.5 keV to 20 keV. The graphene appears “brighter” than the substrate at a low energy of 0.5 keV with a contrast ratio of 9%. At 1.5 keV the monolayer graphene exhibits a similar SE intensity to the substrate, i.e. the SE contrast ratio is 1%. The graphene is still discernible since its edge exhibits a visible contrast (7%). At higher energies (>2 keV), the graphene appears “darker” than the substrate (~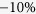). In Fig. 1c, the magnitude of SE contrast (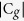) for the monolayer graphene on different substrates (SiO_2_/Si and Au) is sketched as a function of electron beam energy. For the SiO_2_/Si substrate, a contrast maximum (~20%) appears around a beam energy of 5 keV and a minimal contrast (<5%) at c.a. 1.5 keV. On the contrary, the SE contrast of graphene on several substrates (Au in Fig. 1c, Ni and Cu in the supplementary information) is independent of the beam energy, retaining its value (~10%). Both the maximum contrast in SEM and HIM can be larger than that in OM (~5%), making the graphene more identifiable by SE contrast. The key to understanding the contrast variation is the illumination of the supported graphene. Both the primary beam and the backscattered electrons from the substrate can cause SE emission from the graphene. The SE contrast of monolayer graphene, as a function of primary beam energy 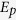 is given by:


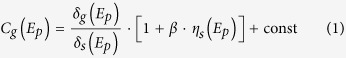


where 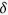
and 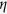 are the SE and BSE yields and the subscripts s and g indicate the substrate and the graphene respectively (see the detailed discussions in section 3 of the supplementary information). 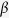 accounts for the lower energy of the substrate BSEs (compared with the primary beam). The SE and BSE yields vary with the beam energy, which can be measured independently (see section 4 of the supplementary information). The calculated result is consistent with the experiments as shown in Fig. 1c. The deviation at low beam energy (<1 keV) is due to the charging of the substrate which is not considered in the calculation and suppresses the SE yield. For the monoatomic metallic substrates, as the beam energy varies the variation in the BSE yield is negligible, which results in the constant graphene contrast.

In the HIM, an insulating substrate will always be charged positively. This positive charging suppresses SE emission by attracting them back into the sample and reducing the landing energy of the primary beam. Without additional compensation mechanisms, the positive charging increases continuously and SE emission will eventually be prohibited when the sample potential is higher than that of the detector. The charged sample appears dark. The charging effect can be compensated by using an electron flood gun which injects electrons into the sample and neutralizes the excess positive charges. However, as the flood gun electrons have an energy of 500 eV they may excite SEs with a yield greater than unity. This means that either inadequate or excessive flooding results in a positively charged surface and reduces the SE emission. Therefore, a properly compensated neutral surface exhibits a maximum brightness and SE contrast, which can be acquired by varying the compensation strength (e.g. electron flood time shown in Fig. 1d). A maximum contrast of ~54% for a single layer of graphene on a SiO_2_/Si substrate was observed at a flood time of 10 μs. We note that the charge compensation does not vary the contrast observed on the non-charging Au substrate since the sample is always electrically grounded. For the insulating substrate, the contrast maximum consistently appears under a set of well-defined imaging parameters (see the optimization of parameters in section 6 of the supplementary information). The results presented hereafter are taken with these parameters.

Figure 2a,b show SE images of few-layer graphene with varying thickness captured in SEM and HIM respectively. The thickness of the graphene was determined by its optical contrast and Raman spectrum prior to the SEM imaging (see section 5 of the supplementary information)^28^,^29^. These images show domains of uniform contrast, which correspond to regions of the same thickness. In Fig. 2c, line-scan profiles of the SE contrast are sketched as a function of lateral locations. The intensity decreases monotonically as the thickness increases and it changes abruptly across the boundary of adjacent domains. The physical boundary of adjacent domains with one-layer thickness difference is atomically sharp, i.e. its lateral extension is at the atomic scale. The boundary revealed in SE images extends ca. 10 nm (see Fig. 2d and section 7 of the supplementary information). Although the value is larger than the physical dimension, it is much smaller than the diffraction-limited value of ~680 nm in an OM. Therefore, the SE contrast provides a much higher lateral spatial resolution than the optical contrast typically used in high-throughput measurement of graphene flake dimensions. This enables us to investigate the properties of graphene that correlate with SE emission at the nanometre scale.

To identify graphene thickness by using SE imaging, the question is whether we can assign a contrast value to a specific thickness, for example, a SE contrast value of ∼−20% in SEM indicating a single layer. To validate this, we need to demonstrate the reliability of the imaging conditions and the reproducibility of the contrast under these conditions. Over 100 individual graphene flakes with different thicknesses varying from 1–10 layers on the SiO_2_/Si substrate were imaged using the same imaging parameters. Figure 2e shows the distributions of the SE contrast (absolute values) have a Gaussian profile with most probable values of (18 ± 1)% and (26 ± 2)% for monolayer and bilayer graphene respectively. The two distributions do not overlap. The absolute values of the maximized contrasts as well as the separation of the two distributions are instrument independent. For example, the two black circle data points in Fig. 2e are the SE contrast of monolayer and bilayer graphene that were obtained in another SEM instrument and they fall in the same intensity range (see detailed discussions in section 8 of the supplementary information). Therefore by using the established criteria, the thickness of a graphene flake can be directly determined by the SE imaging, which is an alternative characterisation method to the optical imaging. Figure 3a summarizes the thickness dependence of the graphene contrast in SEM. As the thickness increases, the contrast decreases. A linear dependence is observed for the thickness range of four to twelve layers, while the contrast decreases more rapidly than the linear relationship for thinner graphene. The rate of contrast reduction becomes slower for thicker layers and the contrast appears to be saturated at about 14 layers.

Based on the consistent SE contrast of graphene, we now demonstrate quantitative extraction of the work function of graphene from SE imaging. The SE intensity *I*_*g*_ collected from the supported graphene is contributed from two sources: the SEs emitted from the graphene and the contribution of the substrate. As mentioned in Equation (1), the SE emission of the graphene was excited by the primary beam *I*_*p*_ and the backscattered electrons from the substrate *η*_*s*_*I*_*p*_. The SEs of the substrate that diffuse through the graphene also contribute to the graphene intensity. The attenuation of the substrate SEs by the graphene depends on graphene thickness and the total reflection at the graphene-vacuum interface. The attenuated substrate contribution exhibits an exponential decrease as layer thickness 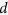 increases, i.e. 
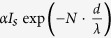
 where 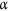
is due to the total reflection, 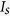 is the SE intensity of the substrate given by *I*_*s*_ = *δ*_*s*_*I*_*p*_, *N* is the graphene layer number, *d* = 0.335 *nm* is the thickness for one layer of graphene and λ is the inelastic mean free path (IMFP) of SEs in graphene. Therefore, the SE contrast for the N-layer graphene is given by:


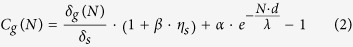


Equation (2) quantitatively describes the experimental results presented in Fig. 3a. The linear contrast decrease for the 4–12 layer graphene indicates 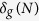 can be regarded as a constant for *N* ≥ 4 and 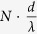 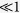. We used the 4–8 layer graphene (blue dashed line in Fig. 3a) to fit the linear contrast decrease and obtained an IMFP value of 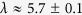 *nm*, which is quite close to the value estimated by the Seah and Dench model^30^. The value of IMFP also explains the slower rate of contrast reduction and the contrast saturation for thicker layers. For the thick graphite (N > 100) a saturated contrast of ~−0.60 ± 0.01 is observed. It corresponds to a situation in Equation (2) that the IMFP is much shorter than the thickness (*N* >* *10), and the substrate attenuation effect can be ignored 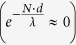. The SE contrast becomes: 
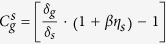
, which gives a similar value of ~ −0.6.

Most importantly, the devation from linearity for graphene thinner than four layers (see Fig. 3a) indicates 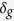 depends on the thickness. The differential of 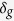 to the SE energy 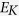, i.e. the SE energy spectrum can be well described by Chung and Everhart’s model^31^:


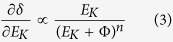


where 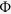 is the work function of graphene. The index number n can be determined as *n* = 4.6 for graphene (see the discussion below). Therefore *δ*_*g*_ is determined by the work function, and we can extract the work function directly from the observed contrast (see section 3 of the supplementary information), which is given by:


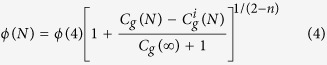


where 
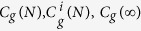
 are the measured graphene contrast, the extrapolated value of the linear relationship and the graphite contrast respectively. 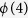 is the work function of four-layer thick graphene and n is given by the SE energy spectrum.

The evolution of work function for few-layer graphene is presented as red squares in the inset of Fig. 3a. The work function of monolayer graphene is determined to be 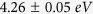. The value increases to 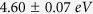 for quad-layer graphene. Similarly, the graphene work function can also be extracted from the He^+^ irradiated SE images (see section 9 of the supplementary information). The work function varies from 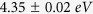 for monolayer to 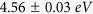 for quad-layer graphene. The two different excitation sources give a similar work function value for each layer, which is consistent with previous reports^6^,^32^,^33^. This supports the quantification developed in our contrast model. Note that since the value of work function becomes a constant for thick graphene (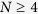), the variation of SE contrast is mainly due to the SE attenuation. This indicates that the extraction of graphene work function from the SE contrast is mainly applicable to few-layer graphene with a thickness up to four layers. The work function map can be calculated from the corresponding SE image, as shown in Fig. 3b. A significant difference in work function can be clearly observed between the thin graphene layers while the difference becomes less distinguishable for thicker layers.

To further verify our model, we measured the SE energy distribution of the sample. Figure 4a shows the measured SE energy spectrum for the freestanding monolayer graphene in SEM with a maximum around 1.5 eV. SE spectra of several few-layer freestanding graphene (monolayer through quad-layer and thicker) were measured and then fitted using the reported work function values (from 4.3 eV for monolayer to 4.6 eV for thicker than quad-layer) and Equation (3). The optimal index number was determined to be 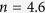 from the fitting results (see the detailed discussions in section 10 of the supplementary information). The fitting of the freestanding monolayer graphene spectrum by Equation (3), with 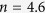, gives a work function value of 4.33 eV. A similar energy spectrum measurement was also carried out in HIM and the fitting index number was determined to be 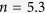 for graphene. Since the measured graphene SE spectrum and its SE contrast were both formed by the partially collected SEs, our work function extraction using Chung and Everhart’s SE distribution equation in Fig. 3 is reliable. The error is less than 0.05 eV.

The measured spectra of the substrate supported graphene also support the validity of our SE contrast model. Figure 4b shows the SE spectrum of monolayer graphene on a SiO_2_/Si substrate, in comparison with the spectra of freestanding monolayer graphene and a SiO_2_ surface. According to our SE contrast model, the SE emission from graphene on a substrate contains the SEs generated within graphene and those which diffuse from the substrate. These two contributions should also be revealed in the SE spectrum of graphene on a substrate. We calculated this SE spectrum using the measured spectra of freestanding monolayer graphene and the SiO_2_ surface, the result is shown as a green dashed line in Fig. 4b. The peak properties (height, position and decay) in the calculated spectrum are similar to that of the peak in the measured spectrum. This similarity supports our contrast model.

The work function variation with number of graphene layers observed in SE imaging is related intimately with the electronic structure of graphene. Figure 4c shows the band structures of the monolayer and quad-layer graphene on a SiO_2_/Si substrate. The band structure of monolayer graphene consists of a simple linear energy dispersion, while thicker graphene has a different band structure. For example, the band structure of bilayer graphene has a parabolic shape, while the band structure of quad-layer graphene becomes more complex and similar to bulk graphite^34^. These band structure differences cause variations in the energy difference between the Fermi level E_F_ and the potential energy E_0_ at infinite distance from the system, i.e. a work function difference. For thicker graphene layers, the higher surface work function will result in a larger energy loss for SEs while approaching the surface. Therefore less SEs can escape from the thicker graphene surface. This is in accordance with the energy spectra measurements in Fig. 4a, in which the spectrum for quad-layer graphene has a lower peak height than that of monolayer graphene, indicating a decrease in SE intensity.

In conclusion, we established a method to quantitatively analyse SE images of graphene on SiO_2_/Si substrates. We have demonstrated that the SE contrast exhibits a universal visibility of few-layer graphene. Graphene with layer numbers of less than ten can be determined from the SE contrast. Besides the quantification of graphene layer thickness, the work function of few-layer graphene can also be quantified by extracting the SE attenuation contributions from the layer-dependent SE contrast. This method provides a high-throughput measurement of graphene’s work function and extends the application fields of SE contrast to quantitative analysis.

## Methods

### Graphene preparation and characterization

Graphene on a highly p-doped SiO_2_/Si substrate was prepared using the micro-mechanical exfoliation method with location and layer numbers determined by optical reflection contrast via an optical microscope (Olympus BX51 with a 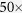 objective lens)^28^. The thickness of the SiO_2_ layer is 285 nm which provides the best optical contrast for imaging. Graphene on metal substrates was prepared by transferring the exfoliated flakes onto the target substrates with the assistance of a Poly-methyl methacrylate (PMMA) film^35^. The substrates were prepared by depositing 50 nm metal films (Au, Cu and Ni) on Si substrates. The freestanding graphene was also prepared by transferring the exfoliated flakes onto a target Si substrate patterned with circular holes of 2 μm in diameter and >10 μm in depth. The PMMA film was then removed in hot acetone (~50 °C) first, then the sample was transferred into hot IPA (~50 °C) and dried in air.

### Collection of SE signal

Secondary electron images of few-layer graphene were obtained from both scanning electron microscopes (Carl Zeiss Supra and FEI Strata DB235) and a helium ion microscope (Carl Zeiss Orion Plus).

In the SEM imaging process the electron beam energy varied from 0.5 keV to 20 keV. A small aperture (10 μm) was used to reduce the beam irradiation damage^36^. The beam current ranged from 5 pA to 20 pA as the beam energy changed. The estimated irradiation dose of each scan varied from ~3 × 10^13^ to ~1.2 × 10^14^ *e*^−^ *cm*^−2^. The emitted SEs were collected by a through-the-lens (in-lens) detector to minimize the influence of back-scattered electrons and chamber generated SEs. The collection parameters were kept constant during the whole experiment (Working distance: 4 mm; Magnification: 2,500×; Resolution: 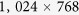 pixel; Scanning time: 26 s). The base chamber vacuum was approximately 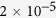 mBar.

In the HIM imaging process, SE images were obtained from a Carl Zeiss Orion Plus scanning helium ion microscope. The He^+^ beam energy was kept constant at 30 keV. The working distance was fixed at 10 mm and the field of view was fixed at 80 μm. An Everhart-Thornley detector collected SEs from graphene with negligible back-scattering ions. The beam current was kept at 1 pA which resulted in an irradiation dose of approximately 5 × 10^11^ ions/cm^2^ for each scan. Only small amounts of defects would be generated at this irradiation dose^37^. When imaging graphene on the SiO_2_/Si substrate, the charge neutralization system (electron flood gun) was always turned on to compensate positive charges introduced by He^+^ irradiation (Flood gun energy: 500 eV; Flood time: 10 μs). The base chamber vacuum was approximately 5 × 10^−7^ mBar.

### Measurement of SE energy spectrum

The measurement of graphene SE energy spectra was carried out using a home-built energy filter in both SEM (Zeiss Supra) and HIM (Zeiss Orion Plus). We placed a TEM grid with periodic holes of 400 μm in diameter on top of the graphene sample. The spacing between graphene and the top of grid was maintained as 0.7 mm. The graphene sample was connected to ground, while a voltage source meter was used to apply a negative voltage from 0 V to 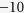 V on the grid. SEs with energy lower than the grid potential were filtered out. By differentiating the measured grid voltage – intensity curve we can obtain the SE spectrum.

## Additional Information

**How to cite this article**: Zhou, Y. *et al.* Quantitative secondary electron imaging for work function extraction at atomic level and layer identification of graphene. *Sci. Rep.* **6**, 21045; doi: 10.1038/srep21045 (2016).

## Supplementary Material

Supplementary Information

## Figures and Tables

**Figure 1:**
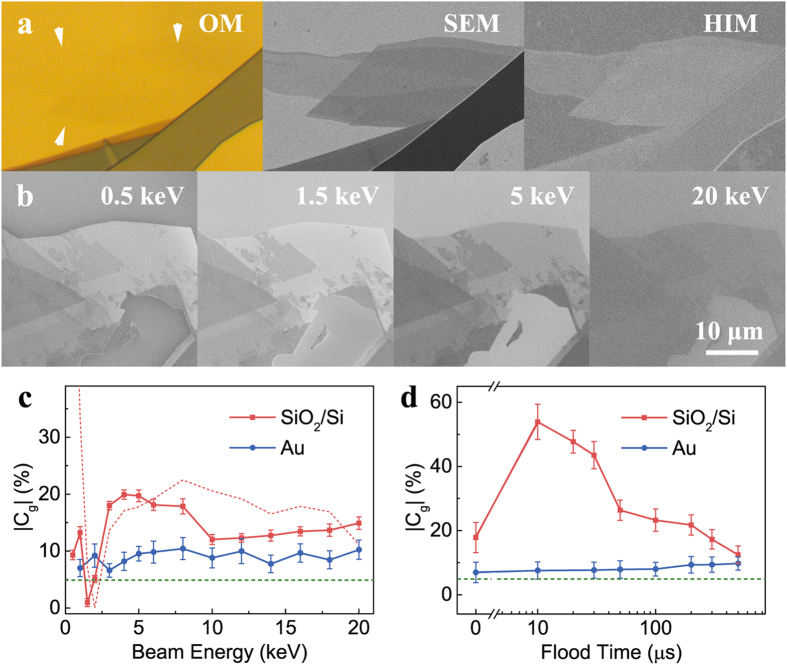
The visibility of graphene in SE contrast. (**a**) Images of a few-layer graphene flake on a Au substrate from an optical microscope (OM), a scanning electron microscope (SEM) and a helium ion microscope (HIM) respectively. The graphene is almost invisible in the optical images, its position is marked by the white arrows. However, the same graphene flake can be clearly observed in SEM (its position is also marked by the white arrows) and HIM images. (**b**) A series of SE images of graphene on a highly p-doped Si substrate covered with 285 nm SiO_2_ (SiO_2_/Si) irradiated with different electron beam energies of 0.5 keV, 1.5 keV, 5 keV and 20 keV. The monolayer graphene is almost invisible with a 1.5 keV electron beam but is visible at all the other beam energies. (**c**) The SE contrast of monolayer graphene on SiO_2_/Si (red squares) and Au (blue circles) substrates under different electron beam energies. The red dashed line shows the calculated SE contrast of monolayer graphene on SiO_2_/Si substrate using Equation (1). (**d**) The SE contrast of monolayer graphene on SiO_2_/Si (red squares) and Au (blue circles) substrates with a different charge neutralization parameter (flood time) in HIM. The value of optical contrast (~5%) is drawn as a green dashed line in both figures for comparison. The error bars in Fig. 1c,d were calculated using the errors of corresponding measured image grey value intensities.

**Figure 2:**
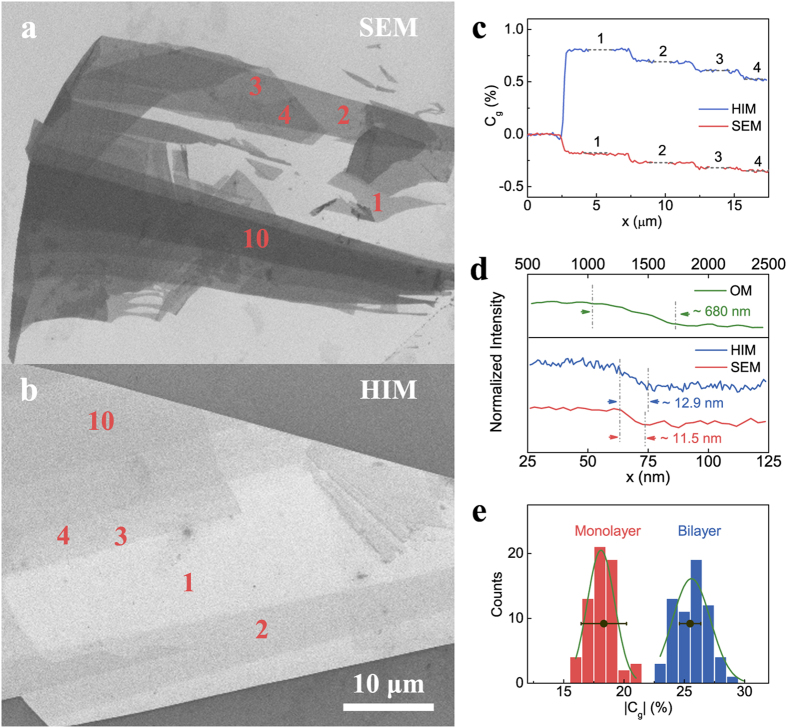
Graphene layer identification from SE contrast. (**a**) A SE image of few-layer graphene under 5 keV electron beam irradiation (**b**) A SE image of few-layer graphene under 30 keV He^+^ irradiation. The location of thin (monolayer to quad-layer) and thick (10-layer) graphene layers are marked by the numbers in the two images. (**c)** Line profiles of SE contrast for the 1–4 layer graphene measured from Fig. 2a,b. Different graphene layer thickness corresponds to the distinct intensities that can be identified. (**d**) High resolution line intensity profiles across the monolayer (right side) and bilayer (left side) graphene edge in three different microscopes: OM, SEM and HIM. The intensities are normalized by the left (brighter) side of bilayer graphene. The measured graphene edge width in OM is ~680 nm, much larger than that in SEM and HIM. (**e**) Normalized intensity statistics of the monolayer and bilayer graphene under 5 keV electron beam irradiation. Both graphene layers obey the Gaussian distribution (green curves) with two peaks separated from each other. The two black circles with error bars show the contrast values of monolayer and bilayer graphene measured in a different SEM. Close values were obtained for the same layer thickness.

**Figure 3:**
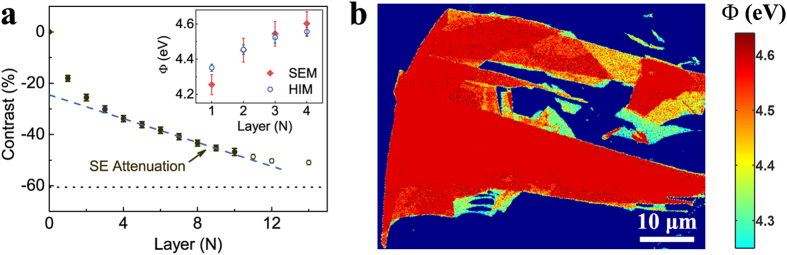
Extraction of graphene work function from the SE contrast. (**a)** SE contrast for the 1–14 layer graphene under 5 keV electron beam irradiation. The relative SE intensities for 11–14 layer graphene were obtained from individual samples and marked as open black circles. The blue dashed line is the linear fitting to the 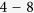 layer graphene, which represents the attenuation effect of SEs from thick graphene 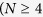) surface. The deviation from the linear fitting for the 1–3 layer graphene layers corresponds to the work function variation from the saturated value of 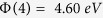. The work function for monolayer graphene is extracted to be 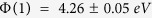. The error bars were obtained from the deviations of measured intensity values. The dot line shows the saturated contrast (∼−60%) value for very thick graphite. The inset figure shows the dependence of graphene work function Φ extracted from 5 keV electron beam imaging (red squares) and 30 keV He^+^ beam imaging (blue open circles). The error bars were obtained from both the SE intensity errors and the linear fitting errors in Fig. 3a. (**b**) A work function map of a graphene flake, which was transformed from the SE image of Fig. 2a. The cyan coloured area has a lowest work function around 4.3 eV while the red coloured area has a highest work function around 4.6 eV.

**Figure 4:**
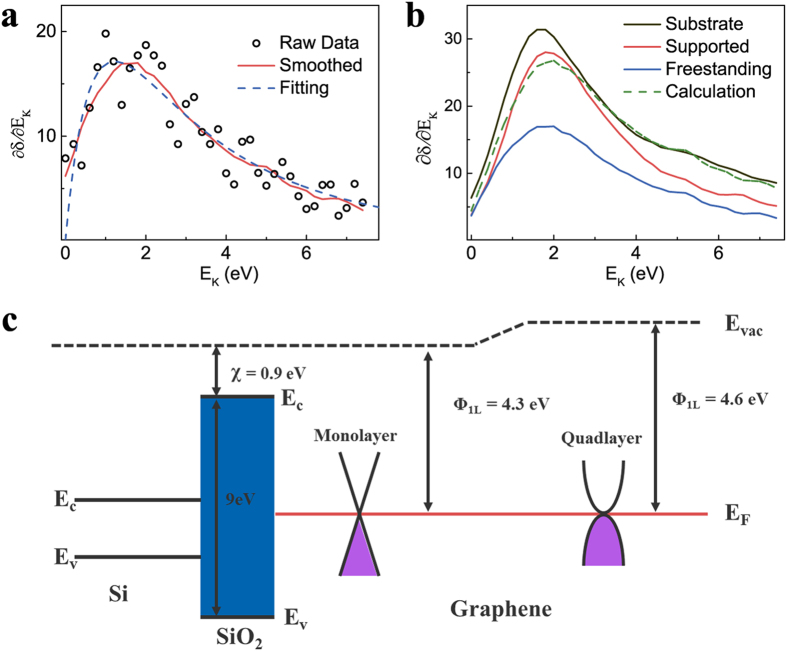
Validity of the SE contrast model and work function extraction. (**a**) SE energy spectrum for freestanding monolayer graphene. The result contains raw data (black open circles), smoothed spectrum (red line) and spectrum fitted by Chung and Everhart model (blue dashed line). The spectrum exhibits a maximum peak around a SE energy of 1.5 eV and can be well fitted by Equation (3) with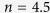. The fitting value of work function for the freestanding monolayer graphene is 4.33 eV. (**b**) SE spectrum of the substrate supported monolayer graphene (red line), in comparison with the spectra of freestanding monolayer graphene (blue line) and the substrate (black line). The green dashed line shows the spectrum calculated by our SE contrast model. (**c)** Band structure diagram of graphene on a SiO_2_/Si substrate. The band structures of the Si substrate, SiOayer, monolayer and quad-layer graphene are shown in the diagram from left to right.
