# Supplementary material for: Quantitative secondary electron imaging for work function extraction at atomic level and layer identification of graphene
Source: Sci Rep. 2016 Feb 16;6:21045. doi: 10.1038/srep21045 (PMC4754635; doi:10.1038/srep21045)
Supplement: Supplementary Information [file srep21045-s1.pdf]

Supporting Information

Quantitative secondary electron imaging for work function extraction at atomic level and layer identification of graphene

Yangbo Zhou[1], Daniel S Fox[1], Pierce Maguire[1], Robert O'Connell[1], Robert Masters[2], Cornelia Rodenburg[2], Hanchun Wu[3], Maurizio Dapor[4], Ying Chen[5], and Hongzhou Zhang[1*]

[1]*School of Physics and CRANN, Trinity College Dublin, Dublin 2, Ireland*

[2]*Department of Materials Science and Engineering, the University of Sheffield, United Kingdom*

[3]*School of Physics, Beijing Institute of Technology, Beijing, 100081, People's Republic of China*

[4]*European Centre for Theoretical Studies in Nuclear Physics and Related Areas, Via Sommarive, 18 - I-38123 Povo, Trento, Italy*

[5]*Institute for Frontier Materials, Deakin University, Waurn Ponds, VIC 3216, Australia*

*Correspondence and request for materials should be addressed to Hongzhou Zhang (hozhang@tcd.ie)

1. Optical and secondary electron (SE) visibility of graphene on metal substrates

Figure S1.1 shows optical and SE images of graphene on two different metal substrates of Cu and Ni. The pristine graphene flakes were exfoliated onto a Si substrate with 285 nm thickness of SiO_2 (SiO_2/Si), which exhibited a visible optical contrast that can be observed under an optical microscope (Figures S1.1a and S1.1d). After being transferred onto metal substrates, their optical contrast turned to be much lower than those on SiO_2/Si substrate. For few-layer graphene flakes on both the Cu (Figure S1.1b) and Ni (Figure S1.1e) substrates, they are almost invisible under an optical microscope. On the contrary, the SE images taken by a 5 keV electron beam still show a visible contrast that the few-layer graphene flakes can be clearly observed (Figures S1.1d and S1.1f). The measured SE contrast for monolayer graphene on these two substrates were (7 ± 1) % for Cu and (9 ± 2) %, which are quite close to the value of (10 ± 2) % for monolayer graphene on Au shown in Figure 1a.

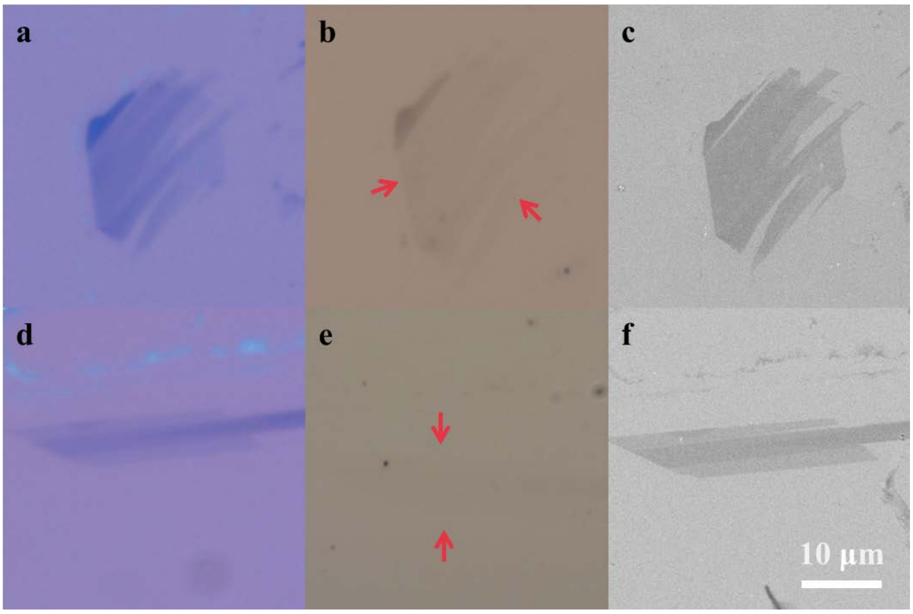

Figure S1.1| Optical and SEM images of few-layer graphene on metal substrates. a, an optical image of a few-layer graphene flake on a Si substrate with 285 nm thickness of SiO_2 (SiO_2/Si). **b,** the optical image of selected graphene flake after transferred onto a Cu substrate, its existence is marked by two red arrows. **c**, the corresponding SE image taken under a 5 keV electron beam irradiation. **d,** an optical image of a few-layer graphene flake on the SiO_2/Si substrate before transferring onto the Ni substrate. **e,** the optical image of selected graphene flake after transferred onto a Ni substrate. The two red arrows shows the region of transferred graphene, which is almost invisible under the optical image. **f**, the corresponding SE image taken under a 5 keV electron beam irradiation.

2. Calculation of graphene optical contrast on a supported substrate

Figure S2.1a shows a schematic of light reflection from a graphene sheet on a single layer structured substrate (e.g. metals). The reflected light from graphene surface has two contributions: one is the direct reflection of incident light on air/graphene interface; the other is that the incident light first refracts into graphene layer, then reflect at the graphene/substrate interface and finally refracts back into air. Assuming the incident light is perpendicular to the graphene plane, the total reflected light intensity that is described by Fresnel's raw can be written is:

$$I(n_1) = |(r_1 e^{i\Phi_1} + r_2 e^{-i\Phi_1}) \times (e^{i\Phi_1} + r_1 r_2 e^{-i\Phi_1})^{-1}|^2 \text{ (2.1)}$$

where

$$r_1 = \frac{n_0 - n_1}{n_0 + n_1}$$

$$r_2 = \frac{n_1 - n_2}{n_1 + n_2}$$

are the relative indices of refraction. n_0, n_1 and n_2 are the reflective index of air, graphene and substrate respectively. $\Phi_1 = 2\pi n_1 d_1/\lambda$ is the phase shift due to changes in the optical path within graphene layer. For the more complex graphene/SiO_2/Si trilayer structures, there will be an additional light reflection at the SiO_2/Si interface. Therefore the total reflected light from graphene surface is given by:

$$I(n_1) = \left|\frac{r_1 e^{i(\Phi_1+\Phi_2)} + r_2 e^{-i(\Phi_1-\Phi_2)} + r_3 e^{-i(\Phi_1+\Phi_2)} + r_1 r_2 r_3 e^{i(\Phi_1-\Phi_2)}}{e^{i(\Phi_1+\Phi_2)} + r_1 r_2 e^{-i(\Phi_1-\Phi_2)} + r_1 r_3 e^{-i(\Phi_1+\Phi_2)} + r_2 r_3 e^{i(\Phi_1-\Phi_2)}}\right|^2 \text{ (2.2)}$$

where $r_3 = \frac{n_2 - n_3}{n_2 + n_3}$, n_2 and n_3 is the reflective index of SiO_2 and Si layer respectively. $\Phi_2 = 2\pi n_2 d_2/\lambda$ is the phase shift due to changes in the optical path within SiO_2 layer, d_2 is the thickness of SiO_2 layer. The optical contrast C is defined as the relative intensity of reflected light in the presence $(n_1 \neq 1)$ and absence $(n_1 = n_0 = 1)$ of graphene,

$C = 1 - \frac{I(n_1)}{I(n_1=1)}$ (2.3)

The monolayer graphene has a layer thickness of d_1= 0.335 nm. It has been reported that the few-layer graphene has the same reflective index as the bulk graphite[1]. The reflective index values of both graphene and metals of substrate are wavelength dependent. We obtained their values from an online database (http://refractiveindex.info), and used matlab (R2012b) to calculate the optical contrast. The results are shown in Figure S2.1b. All three measured metal substrate has a low optical contrast of ~ 2 % for the monolayer graphene in the visible wavelength range from 400 nm to 700 nm. The contrast values change little at different wavelength of incident light. The calculation results also show that the Ni substrate will result in a lower optical contrast for monolayer graphene than the Au and Cu substrate, while the Si substrate with 285 nm thickness of SiO_2 provides a much better (5 times larger) optical contrast than all three metal substrates, which meets well with our experimental observations.

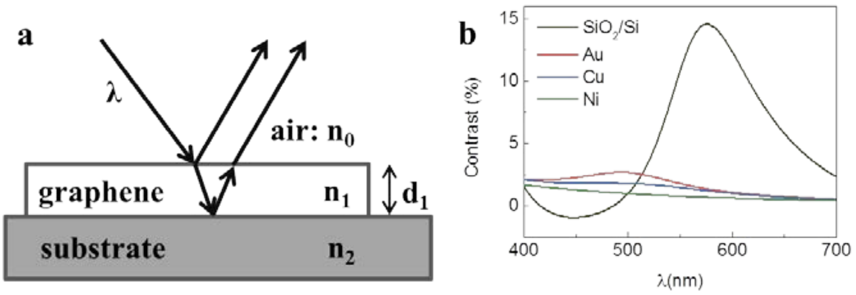

Figure S2.1| Calculated optical contrast of graphene. a, a schematic of light reflection from a graphene sheet on a single-layer structured substrate. **b**, Calculated contrast of monolayer graphene on different substrates of SiO_2/Si, Au, Cu and Ni.

3. Calculation of graphene SE contrast on a supported substrate

In this section, we will present a physical model to calculate the SE contrast of graphene on a supported substrate, and discuss its dependence with beam energy and layer thickness. The irradiation of primary electrons (PEs) and generation of SEs are shown in Figure S3.1. A few-layer graphene flake is placed on the surface of a bulk substrate and irradiated by a high energy PE beam. The PEs can penetrate through the thin graphene layers and goes deeply into the substrate before they lose energy. SEs and back-scattered electrons (BSEs) will first be produced within the graphene layer during the PE penetration process, and escape from the graphene surface. The substrate can also generate SEs and BSEs. These electrons will diffuse into the thin graphene layer and escape from the graphene surface. The attenuation of these electrons might also excite SEs within the thin graphene layer by losing energies. The escaped SEs and BSEs might hit the chamber and generate a background SE signal. The total signal that is collected by a SE detector will contain the contribution from all these generated electrons.

In our experiments, we mainly focus on the SE emissions from thin graphene flakes. The measured flakes usually have the layer number N of $N \leq 10$. We can make reasonable assumptions to ignore the signals from some parts of generated electrons in the total collected signal. Firstly, we ignore the energy loss of the PE beam when penetrating through the graphene layer. The Bethe theory estimate that the stopping power of a 5 keV electron in graphite is approximately 7 eV/nm[2]. Therefore the energy loss through a few-layer graphene ($N \leq 10$) is less than 1 %. The energy and intensity of the beam that enters the substrate can be regarded as the same as the primary incident beam. Secondly, we ignore the contribution of all BSEs. BSEs generated in graphene

can be ignored due to its ultralow yield. Our simulation (using Casino v 2.48) shows that even for the 10 layer graphene, the BSE yield is below 0.001 at about 5 keV. Although the BSE yield of substrate cannot be ignored (e.g. about 0.1 for the SiO_2/Si substrate at a 5 keV beam energy), however, the through-the-lens (in-lens) SE detector only collects electrons with small emission angles. The maximum collection angle in our experiment is approximate 26 (working distance 4 mm, lens hole diameter 4mm),therefore the collected BSEs is < 0.02 % and can be ignored. Furthermore, we can ignore the SEs excited by substrate generated SEs. This is because the minimal ionization energy of graphite is 11.26 eV, while from the SE spectrum over 80 % of the attenuated SEs has a low energy below 10 eV. Even a SE is excited, the substrate SEs will lose energy and might not escape from the graphene surface. We estimated the contribution of background signal to be less than 10 % and it can also be ignored. Therefore, the collected SE current intensity from a graphene surface(I_g) contains signals that mainly come from: (a) SEs generated by the PE beam (intensity of: $I_p\delta_g(N)$, where I_p is the current of is the current of the PE beam, $\delta_g(N)$ is the SE yield of the thin graphene with layer number of N). (b) SEs generated by substrate BSEs (intensity of: $I_p\eta_s\beta\delta_g(N)$, where η_s is the BSE yield of substrate, β refers to how much more SEs are excited by mean per BSE than per PE. Our simulation give ≅ 1.8 .) (c) SEs attenuated from substrate (intensity of: $I_p\delta_s\alpha e^{-Nd/\lambda}$, where δ_s is the SE yield of the substrate, α is a coefficient that describes how much proportion of SEs escape from the graphene surface to those from the substrate surface due to the angular distribution of SEs, d is the thickness for a monolayer graphene, λ is the mean inelastic mean free path (IMFP) of attenuated SEs.

We assume all the escaped SEs can be collected efficiently by the SE detector. The total intensities from graphene (I_g) and substrate (I_s) surface are given by:

$I_g(N) \cong I_p \cdot \delta_g(N) \cdot (1 + \beta\eta_s) + \delta_g(N) + I_p \cdot \delta_s \cdot \alpha \cdot e^{-\frac{Nd}{\lambda}}$] (3.1)

$I_s \cong I_p \cdot \delta_s$ (3.2)

The graphene SE contrast is defined as:

$C_g(N) = \frac{I_g - I_s}{I_s} = \frac{\delta_g(N)}{\delta_s}[1 + \beta\eta_s] + \alpha e^{-\frac{Nd}{\lambda}} - 1$ (3.3)

We first discuss the relationship of monolayer SE contrast with the beam energy, as we presented in Figure 1c. For monolayer graphene we have N=1. We ignore the SE attenuation in the monolayer graphene (i.e. $e^{-\frac{d}{\lambda}} \cong 1$) The SE contrast becomes:

$C_g(E_P) = \frac{\delta_g(E_P)}{\delta_s(E_P)}[1 + \beta\eta_s(E_P)] + \alpha - 1$ (3.4)

We can obtain the ratio of $\frac{\delta_g(E_p)}{\delta_s(E_p)}$ from experiment measurements (see next section for the discussion of SE yield). η_s can be computed from the simulation using Casino software (version 2.48). The energy distribution of SEs can also be obtained, then β can be calculated to have a value ~ 1.8 for high energy electrons (> 2 keV). We calculated α to be ~ 0.3 by assuming the SEs overcome the surface affinity to escape from the SiO_2 surface and into the graphene/SiO_2 gap first, then penetrate into graphene. It is an underestimated value because at such a small gap the SEs might directly diffuse from the substrate into graphene without energy loss. We found that larger value of α=0.45 to fits the results better in Figure 3c. For graphene on a metal substrate (e.g. Au), $\frac{\delta_g(E_p)}{\delta_s(E_p)}$ will be a constant because the obey the same SE energy distribution. Our simulation also shows that backscattering coefficient of Au doesn't greatly change with beam

energy (always around 44% at energy > 1 keV). This will result in a constant contrast at different beam energies.

We then discuss the layer dependence of graphene SE contrast. From the measured SE spectra in Figure 4 we can estimate the most probable energy of escaped SEs is ~ 1.5 eV. SEs in graphene have to overcome a surface potential barrier (i.e. work function of ~4.3 eV). Therefore the mean energy of escaped SEs in graphene can be estimated to be ~6 eV. The IMFP value for a 6 eV electron can be roughly estimated by the Seah and Dench model[3]

$$\lambda(E) = a\frac{538}{(E-E_F)^2} + 0.41a(E-E_F)^{1/2} \quad (3.5)$$

Where a is the thickness of graphene monolayer. The model gives a value of ~6 nm. This value is much larger than the graphene layer thickness. Therefore we use a linear approximation of: $e^{-Nd/\lambda} \cong 1 - Nd/\lambda$, for $Nd/\lambda \ll 1$. Eq.(3.3) can be written as:

$$C_g(N) \cong -\alpha\frac{d}{\lambda} \times N + [\alpha + \frac{\delta_g(N)}{\delta_s}(1+\beta\eta_s) - 1] \quad (3.6)$$

Here $\delta_g(N)$ can be computed by the integration of the graphene SE energy distribution:

$$\delta_g(N) \propto \int_0^{50\,eV} \frac{E_K}{\left(E_K+\Phi(N)\right)^n} dE_K = \frac{[\Phi(N)]^{2-n}}{(n-1)(n-2)} \quad (3.7)$$

Where $\Phi(N)$ is the work function for graphene with thickness of N layers. We assume $\Phi(N)$ has a constant value for $N \geq 4$. Previous reports gives that Φ increases from 4.3 eV for N=1 to 4.6 eV for $N \geq 4$[4], The coefficient α can be computed by considering how many SEs that can diffuse from substrate to graphene and escape from the surface. This is determined by the SE angular distribution at the surface. The SE emission at the specimen/vacuum interface is governed by a refraction effect[5], which is given by:

$\sqrt{E_s}\sin\theta_s = \sqrt{E_k}\sin\theta_k$ (3.8)

Where E_s and E_k are the kinetic energies of SEs in the specimen and in vacuum, θ_s and θ_k are the corresponding incident and refracted angles. We ignore the energy loss for a SE to diffuse from the SiO_2 substrate into graphene, therefore the energy that a SE need to overcome and escape from graphene surface is $\Phi - \chi$, where χ is the affinity of SiO_2 layer ($\chi \cong 1$eV). The maximum escape angle for a SE with energy E_K is: $\theta_m = cos^{-1}\sqrt{\frac{\Phi-\chi}{E_K+\Phi-\chi}}$, α can be computed as:

$\alpha(N) = \int_0^{50\ eV}\int_0^{\theta_m}\frac{d\theta}{\pi/2}dE_K$ (3.9)

From Eq. (3.8) we can estimate α to be a constant of ~ 0.4.

Our experiments show a linear contrast decreasing behavior for $4 \leq N \leq 12$. A large deviation can be observed for the few graphene layers for $1 \leq N \leq 3$. The observation can be attributed to the variation of graphene work function with the layer thickness. For $4 \leq N \leq 12$, the work function will become a constant value and determined as $\Phi(4)$. The linear response corresponds can be described Eq. (3.6) with the constant work function value of $\Phi(4)$:

$C'_g(N) = -\alpha\frac{d}{\lambda} \times N + [\alpha + \frac{\delta_g(4)}{\delta_s}(1 + \beta\eta_s) - 1]$ (3.10)

The difference of values between Eq.(3.6) and Eq.(3.10) shows the deviation from linear contrast decrease that we observed.

$C_g(N) - C'_g(N) = \frac{\delta_g(N)-\delta_g(4)}{\delta_s}(1 + \beta\eta_s)$ (3.11)

When $N \rightarrow \infty$, Eq. (3.6) corresponds to the SE contrast of a bulk graphite. We assume the BSE yield doesn't change, Eq. (3.6) then becomes:

$C_g(\infty) = \frac{\delta_g(4)}{\delta_s}(1 + \beta\eta_s) - 1$ (3.12)

We assume the graphene SE yield is independent of the layer thickness for few layers, therefore according to Eq.(3.7) there is $\frac{\delta_g(N)}{\delta_g(4)} = [\frac{\Phi(N)}{\Phi(4)}]^{2-n}$. The work function of few-layer graphene ($1 \leq N \leq 3$) can be written as:

$$\phi(N) = \phi(4)\left[1 + \frac{C_g(N) - C_g^i(N)}{C_g(\infty)+1}\right]^{1/(2-n)} \quad (3.13)$$

Therefore the work function for a N-layer graphene ($1 \leq N \leq 3$) can be extracted using the measured values of its SE contrast ($C_g(N)$), linear fitting extension at N-layer ($C'_g(N)$), and the bulk graphite $C_g(\infty)$.

For the He$^+$ irradiation situations, the backscattering yield can be ignored, i.e. $\eta_s \approx 0$. Therefore Eq. (3.3) becomes:

$$C_g^{HIM}(N) = \frac{\delta_g(N)}{\delta_s} + \alpha e^{-\frac{Nd}{\lambda}} - 1 \quad (3.154)$$

The same linear approximation can be applied to the graphene layer SE contrast by He$^+$ irradiation. The same work function extraction formula as Eq. (3.13) can be obtained.

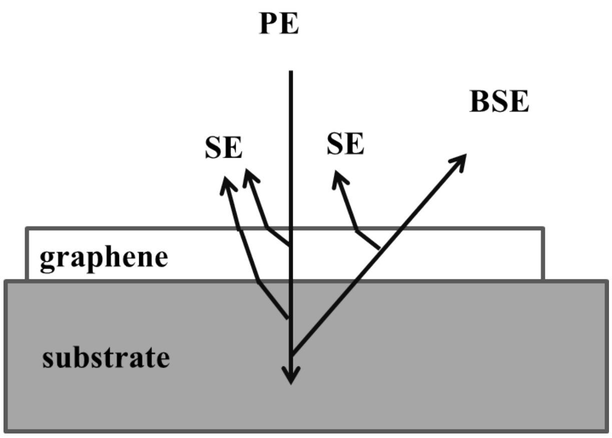

Figure S3.1| A schematic of SE emission from a graphene sheet on a substrate.

4. Evaluation of graphene SE yield

We used a simple method to evaluate the SE yield of the free-standing graphene. Figure S4.1a shows a SEM image of a monolayer graphene on a Si substrate with etched holes (diameter ~ 2 μm, depth > 10 μm). Some holes were covered by the free-standing graphene. We treat the uncovered hole as an ideal Faraday cup because its very deep and very few of SEs and BSEs can escape from the hole. Therefore the primary beam current i_p can be obtained when the hole is irradiated by a primary beam. The current i_g measured from a freestanding graphene will become smaller because the secondary electrons and back-scattered particles will be generated during the beam-graphene interaction process (Fig. S4.1b). Therefore we can obtain the total electron emission yield σ_g of a freestanding graphene, which contains contributions from both SE emission (δ_g) and BSE emission (η_g), and is given by:

$$\sigma_g = \delta_g + \eta_g = 1 - \frac{i_g}{i_p} \quad (4.1)$$

The casino simulation presented in Figure S4.1c shows a low BSE yield below 0.5 % for the e-beam energy higher than 1 keV, which can be ignored. The extracted SE yield is shown as black squares in Figure S4.1d, which has a high value of ~120 % at the low beam energy of ~0.2 keV, and then decreases as beam energy increases. It becomes <10 % when the beam energy is higher than 5 keV. At the same beam energy, the SE yield of SiO_2 surface is around 1[6]. Therefore the SEs generated in the substrate and diffuse into graphene dominate the SE emission. For the HIM irradiation process, the back-scattered ion coefficient can be calculated by the SRIM simulation, which shows a much smaller value (10^{-5} at the 30 keV He^+ beam energy) and can be ignored. So the total yield we measured could be regarded as the SE yield. The result is shown as red

circles in Figure S4.1d. The SE yield is much higher than that in SEM, and slightly increases with the increase of He^+ beam energy.

For the SE yields of the freestanding graphene and substrate that are used in Equation (1) in the manuscript for SE contrast calculation. We simply measured their grey values in the SE images obtained under the same imaging conditions (working distance of 4 mm, brightness and contrast of in-lens detector settings were 50% and 38% respectively). Their ratio is the value of $\frac{\delta_g(E_p)}{\delta_s(E_p)}$ we used to calculate the SE contrast.

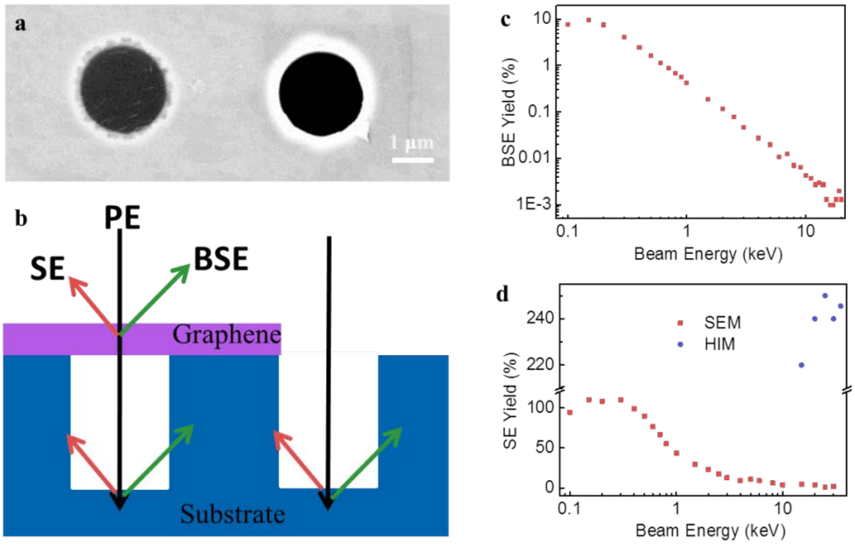

Figure S4.1| **SE yield of a freestanding graphene. a**, A 5 keV SEM image shows the existence of a hole and free-standing graphene. **b**, Diagram of graphene yield measurement. **c**, BSE yield of free-standing monolayer graphene calculated by casino simulation. **d**, SE yield of a monolayer graphene in both SEM and HIM measurements.

5. Determination of graphene layer thickness by optical contrast and Raman spectrum

The optical image of imaged few-layer graphene flakes is shown in Figures S5.1a. The corresponding optical contrast of the red (R), green (G) and blue (B) channels are defined by Eq. (2.3) calculated and presented in Figure S5.1b. . The optical contrast for a N-layer graphene could be fitted by a parabolic relationship[7]. For the green channel with best contrast (~ 5% for monolayer graphene), the relationship is:

$$C(N) = 0.0954 + 0.04615 \times N - 0.0169 \times N^2 \quad (5.1)$$

Therefore the optical contrast could determine the thickness information of few layer graphene. We also used Raman spectrum to confirm the thickness information of few-layer graphene, as shown in Figure S5c. The Raman spectrum of a monolayer graphene has a different intensity ratio of G peak (~1580 cm^{-1}) to 2D peak (~2680 cm^{-1}) comparing with that of multilayer graphene[8]. However, it is difficult to distinguish the different thicker graphene layers.

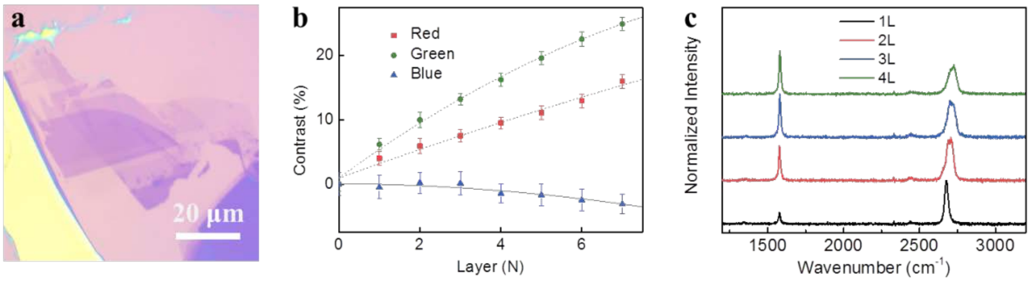

Figure S5.1| Optical contrast of graphene. a, Optical image of a multilayer graphene with layer thickness from 1-7 layers. **b,** Contrast dependence for the multilayer graphene measured in different channels. The red, green, blue data points represent for red, green, blue channels respectively. Black curves represent the parabolic fit to the contrast for three channels. **c,** Raman spectra of monolayer (1L), bilayer (2L), trilayer (3L) and quad-layer graphene (4L) graphene.

6. Optimisation of the SE imaging conditions in SEM

The influence of primary beam energy and substrate to the SE contrast have been discussed in the manuscript. Here we investigate the other parameters that might have potential influence. All the experiments were done in the Zeiss Supra SEM.

We first varied the working distance at a constant beam energy of 5 keV in the Zeiss Supra SEM. The SE intensity variation of both substrate and different graphene layers is shown in Figure S6.1a. As the working distance increases from 3 mm to 7 mm, the SE intensities of substrate and graphene layers increase first then decrease. The maximum intensity is determined as a working distance between 4 to 5 mm. The variation can be explained as the change of SE collection efficiency by the in-lens detector, and the highest efficiency happens at 4-5 mm of working distance. The SE intensities for different graphene layers also behaves a different working distance dependence. For the thicker graphene layers, the maximum intensity position slightly shifts to the lower working distance side. The observation can be attributed to the angular distribution of SEs and the work function variations between graphene few layers. The kinetic energies of SEs in graphene and vacuum have a relationship with the material work function Φ: $E_S = E_K + \phi$. The graphene surface working is known to increase with the graphene layer thickness. Therefore for SEs with same kinetic energy E_S in graphene, their energies in vacuum of E_K will decrease. According to Eq. (3.8), the emission angle for escaped SEs will become larger and less collected by the in-lens SE detector. At graphene thickness larger than 4 layers, the work function value no longer changes, thus the emission angle does not change.

Figure S6.1b shows the SE contrast of graphene with the working distance. one can see that around the optimized working distance (4-5 mm) the graphene contrast remains

almost as a constant. Therefore the graphene work function extraction based on our contrast model is not sensitive to the working distance, i.e. the SE collection efficiency. However, we still imaged all graphene flakes at the optimized condition, so that SEs could be collected quite efficiently.

We also investigated the influence of the other imaging parameters, such as scan speed and beam current. Figure S6.2 shows the SE images taken at the different scanning speed of 1, 2, 4 and 8. The corresponding scanning time per frame is 122 ms, 220 ms, 731 ms and 10.2 s respectively. The image quality, i.e the signal to noise greatly improves, meanwhile the SE contrast remains as a constant at short scanning time but increases at a longer scanning time, as shown in Figure S6.3a. The decrease of the graphene contrast is due to the substrate charging at the longer scanning time. The variation of SE contrast with the beam current is shown in Figure S6.3b. The beam current is controlled by the aperture, which varies from 7 μm to 30 μm. The graphene SE contrast also slightly decreases as the beam current increases. Therefore the scan speed and beam current will not greatly influence SE contrast.

As a conclusion, the working distance will strongly affects the SE contrast while the scan speed and beam current only slightly changes to the SE contrast. In our experiment, we select the working distance to be 4 mm to obtain a best contrast.

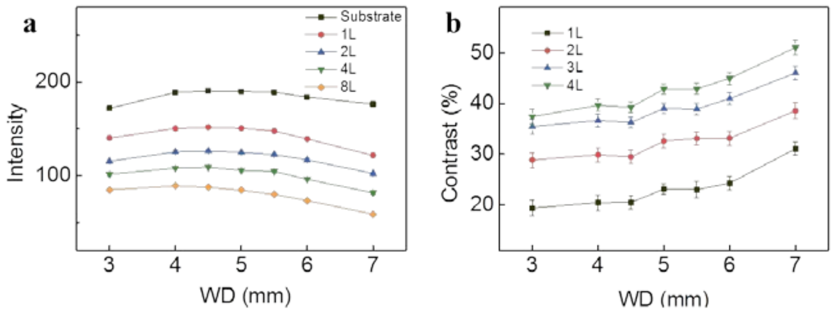

Figure S6.1| The influence of SE imaging by varying working distance (WD). a-b shows the variation of SE intensity and SE contrast with the change of working distance.

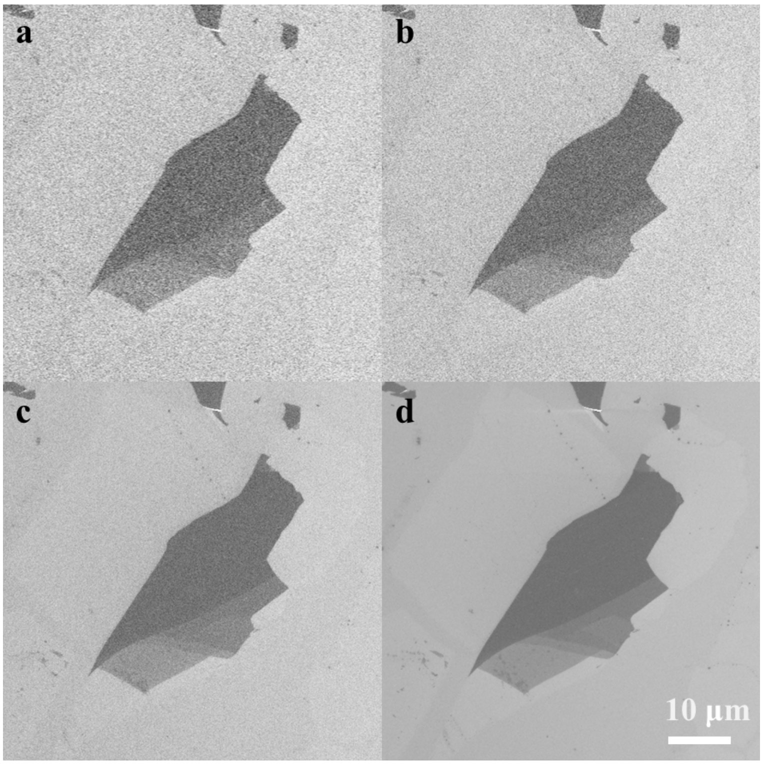

Figure S6.2| SE image of a graphene flake under different scanning speed. a-d has the different pixel dwell time of 122 ms, 220 ms, 731 ms and 10.2 s respectively

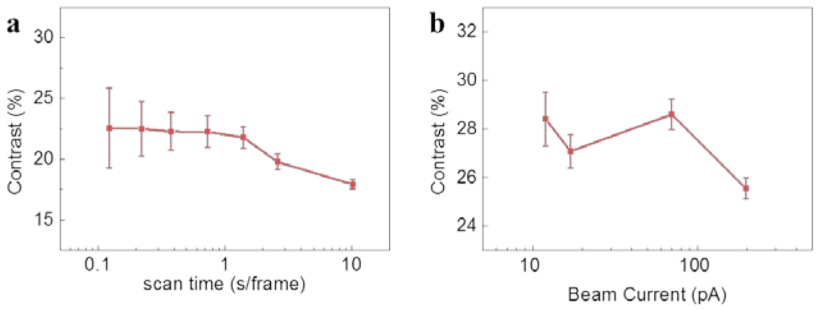

Figure S6.3| Variation of graphene SE contrast by varying different parameters. a, the scan speed changes. b, the beam current changes.

7. High resolution SE images of graphene edges

Figure S7.1 shows the SEM, HIM and optical images of edges between the monolayer and bilayer graphene. The red arrows show the edges between monolayer to bilayer graphene which are used to obtain the line intensity profile in Figure 1d. Optical image gives a lower spatial resolution than SE images.

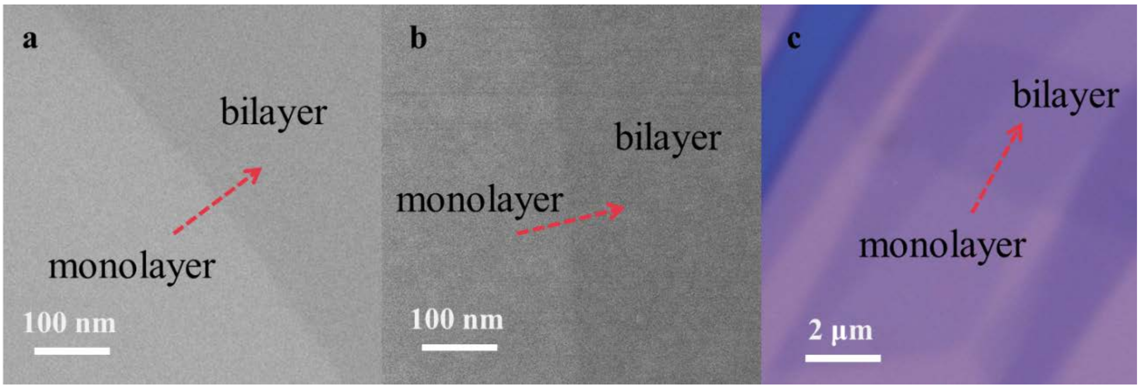

Figure S7.1| High resolution images of graphene edges between monolayer and bilayer graphene. a, a SEM image. **b**, a HIM image. **c**, an optical image.

8. Statistics to graphene SE contrast

We investigated the graphene SE contrast in different microscopes. These microscopes include a Carl Zeiss Supra SEM with the in-lens detector, a FEI Strata DB235 SEM with a through-the-lens(TLD) detector which is similar as the in-lens detector in collecting SEs, and a Carl Zeiss Orion Plus Helium ion microscope with a different excitation source.

The main contrast statistical experiments were proceeded in the Carl Zeiss Ultra SEM with the 5 keV beam energy and 4 mm working distance. We measured 158 graphene samples in total. The measured samples included 60 monolayer, 62 bilayer, 22 trilayer and 14 quad-layer graphene flakes. The distributions of corresponding SE contrast are shown in Figure S8.1. The SE contrast of each graphene layer obey the Gaussian distribution, indicating that each graphene layer will have a certain contrast value.

We also measured 8 of these samples in another SEM (FEI Strata D235). The parameters (beam energy, working distance, etc.) were kept as the same. The results were shown in Table S8.1. The two different microscopes gave the similar SE contrast on the same samples.

The statistical result of graphene contrast in HIM is shown in Table S8.2. We measured 10 individual graphene samples in total, which included 6 monolayer, 2 bilayer and 3 trilayer flakes. The imaging parameters were selected as 30 keV primary beam energy, 500 eV flood energy with 50 μs flood time to generate a charge neutralized state .The average contrast for the three different graphene layers are around 0.17, 0.13 and 0.09 respectively. Therefore the same graphene layer thickness also reflects the same SE intensity at the charge neutralized state in HIM.

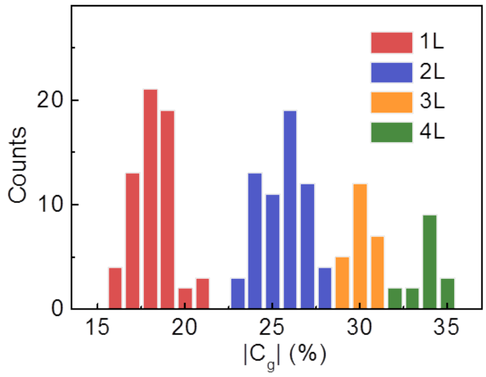

Figure S8.1| Contrast statistics of different graphene flakes. All the samples were imaged under the irradiation of a 5 keV electron beam. 60 monolayer, 62 bilayer, 22 trilayer and 14 quad-layer graphene samples were measured.

Sample	Layer	Contrast	
		Zeiss Supra	FEI Strata
1	2	0.27	0.25
2	1	0.19	0.19
3	2	0.26	0.24
4	2	0.27	0.23
5	1	0.19	0.17
6	1	0.19	0.17
7	3	0.35	0.29
8	4	0.35	0.30

Table S8.1| *Contrast measurement of the same graphene flakes in two different SEMs (Zeiss Ultra and FEI Strata DB 235)*

Sample	Layer	Contrast
1	1	0.16
2	1	0.17
3	1	0.18
4	1	0.15
5	1	0.16
6	1	0.18
7	2	0.13
8	2	0.13
9	3	0.10
10	3	0.08

Table S8.2 *Contrast measurement of graphene flakes in HIM*

9. Layer dependence of graphene SE contrast and work function extraction

We investigated the layer dependence of graphene SE contrast in different situations. The results are shown in Figure S9.1. The few-layer graphene flakes are imaged under the electron beam with different energies (Figure S9.1a), or placed on different metal substrates and imaged by SEM (Figure S9.1b), or imaged by HIM and under the different flood time (Figure S9.1c). The observed layer dependence of SE contrast is similar as we have presented in Figure 3a of the manuscript, i.e. a rapid contrast decrease for the few 1-3 layers followed by an almost linear decrease for thicker layers.

Figures S9.2a and S9.2b show the layer dependence of graphene SE contrast in SEM (5 keV e^- beam) and HIM (30 keV He^+ beam) respectively. The graphene work function is extracted using Eq. (3.13). For the layer dependence of SE contrast in Figure S9.2a, we first obtained the SE contrast values for 1-4 layers of graphene to be: -0.181 ± 0.011, (-0.256 ± 0.013), $(-0.302 \pm 0.013$, -0.338 ± 0.012. The linear fitting to the 4-8 layers gives a slope of -0.0238 ± 0.0004, and the extended linear fitted values for 1-4 layers of graphene to be: -0.266 ± 0.002, -0.290 ± 0.002, -0.314 ± 0.002, -0.337 ± 0.002. The contrast for bulk graphene is measured from a thick graphite flake which exhibits a golden color under an optical microscope, its layer thickness can be estimated to be over 100 layers, the contrast exhibits a saturated value of -0.606 ± 0.013. Therefore according to Eq. (3.13), the work function for 1-4 layer graphene is calculated to be: $4.25 \pm 0.06\, eV$, $4.45 \pm 0.07\, eV$, $4.54 \pm 0.07\, eV$, 4.60 ± 0.07 eV, respectively. From the slope of 4-8 layer linear fitting we can estimate the IMFP from Eq. (3.6).

For the measurements in HIM, the SE contrast of bulk graphite is measured to be 0.435 ± 0.025. The slope of linear fitting is -0.0143 ± 0.0005. We used Eq. (3.13) with n=5.3 and obtained the work function values for 1-4 layer graphene: $4.31 \pm 0.02\ eV$, $4.43 \pm 0.03\ eV$, $4.49 \pm 0.03\ eV$, 4.55 ± 0.03 eV respectively.

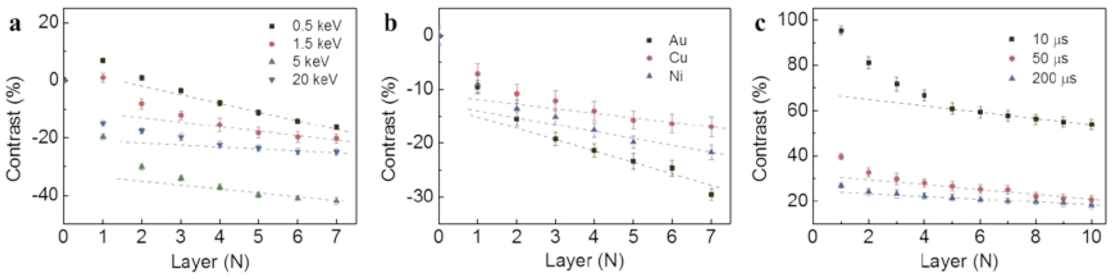

Figure S9.1| Layer dependence of graphene SE contrast. a, graphene on a SiO_2/Si substrate and imaged by SEM with beam energies of 0.5 keV, 1.5 keV, 5 keV and 20 keV. **b,** graphene on different metal substrates (Au, Cu and Ni) and imaged by SEM. c, graphene on a SiO_2/Si substrate and imaged by HIM with different flood time of 10 µs, 50µs and 200µs.

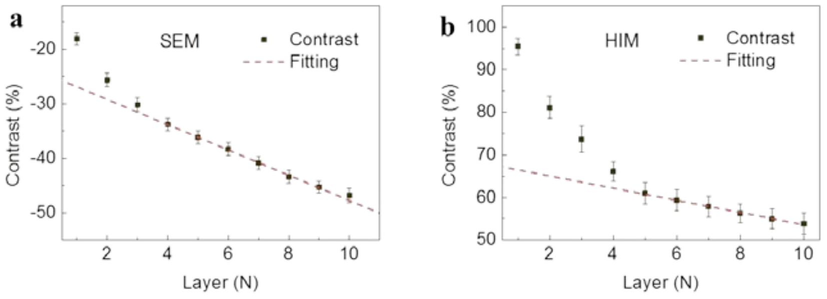

Figure S9.2| Layer dependence of graphene SE contrast with linear fitting for thicker layers in (a) 5 keV SEM and (b) 30 keV HIM.

10. Measurements of graphene SE spectra

Figure S10.1a shows the SEM image of a freestanding monolayer graphene (marked by the red arrow) that is used for SE spectrum measurement. The data we directly obtained from SE energy filtering measurement is a grid voltage bias versus SE intensity curve. Figure S10.1b shows such a curve obtained from a freestanding graphene. The intensity starts to decrease rapidly at an onset point of $V_{onset} \approx -1.6\,V$, indicating SEs starts to be filtered. The relationship between filtered SE energy E_K and grid voltage V_{Bias} is $E_K = -e(V_{Bias} - V_{onset})$. By converting the x-axis in Figure S10.1b to SE energy, and differentiating the filtered SE intensity, the SE spectrum (using Savitzky-Golay Smooth in Origin 9.0, Polynomial Order= 2, Points of Window= 8) of freestanding graphene can be obtained and is shown in Figure S10.1c. To determine the work function from SE spectrum, we convert Eq. (3) to :

$$\sqrt[n]{\frac{E_K}{(\frac{\partial \delta}{\partial E_K})}} \propto (E_K + \Phi) \quad (10.1)$$

We varied n from 3.5 to 5.0, used Eq. (10.1) to fit the spectrum in Figure S10.1c. The work function value could be obtained by the intercept in Eq. (10.1), which changed 2.51 eV to 4.98 eV, as shown in Figure S10.2a. We compared the extracted work function values at different n with reported values (around 4.3 eV), and found that n=4.6 gave the closest value. At n=4.6, the fitting gave a work function value of 4.32 eV.

We measured 5 samples in total. These samples are the monolayer to quadlayer and relatively thick (> 10 layers) freestanding graphene. Their SE energy spectra are shown in Figure S10.2c. For a given index number n, we compared the extracted work function values to the reported work function values (4.3 eV fro monolayer, 4.4 eV for bilayer , 4.5 eV for trilayer and 4.6 eV for quad-layer and thicker layers), and calculated the

average deviations for all the measured 5 samples. The result is shown in Figure S10.2d. For all these measured samples with different layer thickness, n=4.6 gives the closest results to the report work function values.

For the calculation of graphene SE spectrum on SiO_2/Si substrate in Figure 4b, we used the following equation:

$$(\frac{\partial\delta}{\partial E_K})_{cal} = (\frac{\partial\delta}{\partial E_K})_s \cdot \alpha'(E_K) + (\frac{\partial\delta}{\partial E_K})_g \cdot (1+\eta) \quad (10.2)$$

Where the subscripts cal, s and g indicated the calculated result, substrate and graphene respectively, $\alpha' = \frac{\theta_m}{\pi/2}$ describes the proportion of SEs with energy E_K that can escape from the surface, η is the BSE coefficient.

We also measured the SE spectra of graphene in HIM. Figure S10.3a shows the SE spectra of a freestanding monolayer graphene in HIM. Figure S10.3b shows the deviation of extracted work function values from reported value (4.3 eV). n=5.3 gives the closest results.

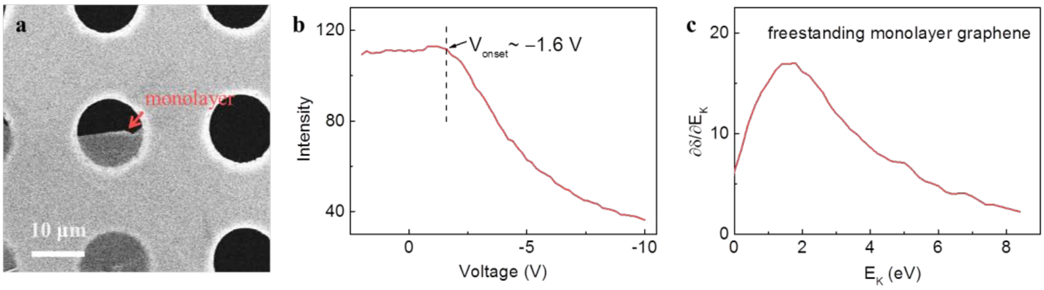

Figure S10.1| SE Spectra of monolayer freestanding graphene. a, SEM image of the measured freestanding monolayer graphene, as marked by the red arrow. **b,** grid voltage vs SE intensity curve from measured flake. **c**, SE spectrum from measured flake by differentiating the curve in (b).

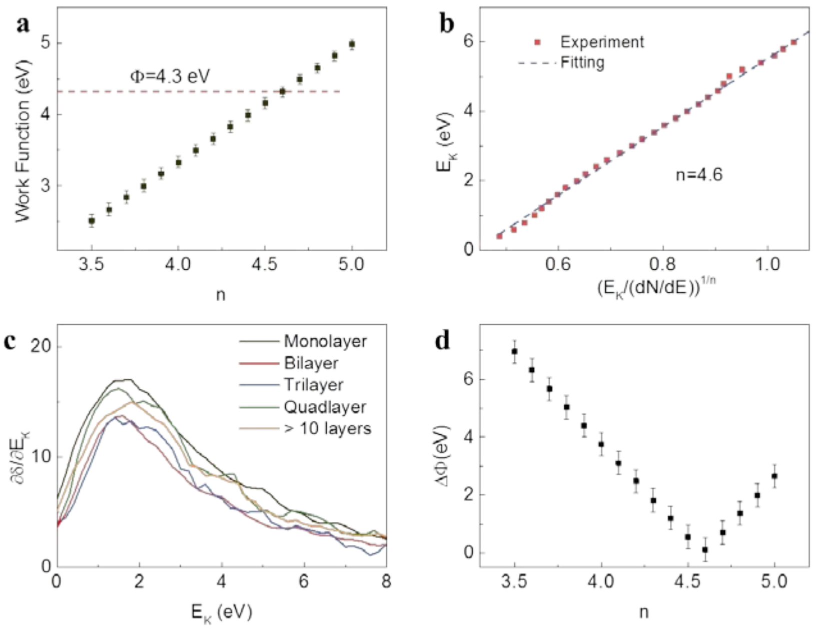

Figure S10.2| Determine of index n. a, Extracted work function values at different n. n=4.6 gives the closet extracted value compared to reported values. **b,** Linear fitting by Eq. (10.1). **c,** SE energy spectra of the monolayer to quadlayer and thick (> 10 layers) freestanding graphene. **d**, Deviation of extracted work function values from reported values at different n. n=4.6 gives the smallest difference between two values.

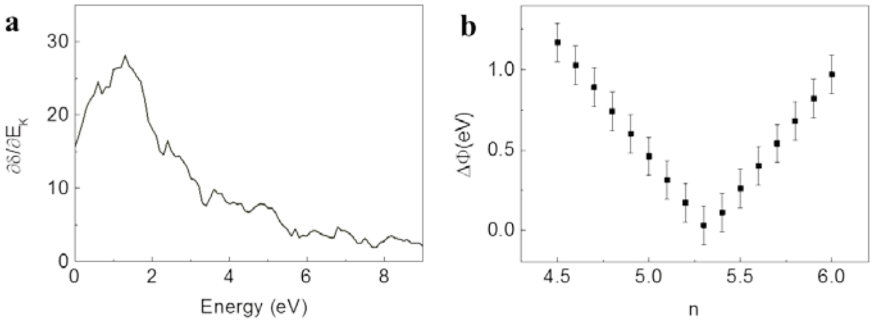

Figure S10.3| SE energy spectrum of graphene in HIM. a, , SE energy spectrum of the monolayer freestanding graphene in HIM. **b**, Deviation of extracted work function values from reported values at different n. n=5.3 gives the smallest difference between two values.

References

1. Ghamsari, B. G.; Tosado, J.; Yamamoto, M.; Fuhrer, M. S.; Anlage, S. M. *arXiv:1210.0575* **2012**.
2. Reimer, L., *Image Formation in Low-Voltage Scanning Electron Microscopy*. SPIE PRESS: 1993; Vol. TT12.
3. Seah, M. P.; Dench, W. A. *Surf. Interface Anal.* **1979,** 1, (1), 2-11.
4. Hibino, H.; Kageshima, H.; Kotsugi, M.; Maeda, F.; Guo, F. Z.; Watanabe, Y. *Physical Review B* **2009,** 79, (12), 125437.
5. Cazaux, J. *Ultramicroscopy* **2010,** 110, (3), 242-253.
6. Yi, W.; Jeong, T.; Yu, S.; Lee, J.; Jin, S.; Heo, J.; Kim, J. M. *Thin Solid Films* **2001,** 397, (1–2), 170-175.
7. Ni, Z. H.; Wang, H. M.; Kasim, J.; Fan, H. M.; Yu, T.; Wu, Y. H.; Feng, Y. P.; Shen, Z. X. *Nano Lett.* **2007,** 7, (9), 2758-2763.
8. Ferrari, A. C.; Meyer, J. C.; Scardaci, V.; Casiraghi, C.; Lazzeri, M.; Mauri, F.; Piscanec, S.; Jiang, D.; Novoselov, K. S.; Roth, S.; Geim, A. K. *Phys. Rev. Lett.* **2006,** 97, (18), 187401.
